# pH-Effect in the Fabrication of ZnO Nanostructured Thin Films by Chemical Bath Deposition for Increasing the Efficiency of Solar Cells

**DOI:** 10.3390/ma16083275

**Published:** 2023-04-21

**Authors:** Abel Garcia-Barrientos, Roberto Carlos Ambrosio-Lazaro, Rafael Ramirez-Bone, Mario A. Garcia-Ramirez, Obed Perez-Cortes, Ruben Tapia-Olvera, Jairo Plaza-Castillo

**Affiliations:** 1 Faculty of Science, Universidad Autónoma de San Luis Potosi, San Luis Potosi 78295, Mexico; 2 Facultad de Electrónica, Benemérita Universidad Autónoma de Puebla, Puebla 72570, Mexico; roberto.ambrosio@correo.buap.mx; 3 Centro de Investigación y de Estudios Avanzados del IPN, Unidad Querétaro, Queretaro 76001, Mexico; rrbon@cinvestav.mx; 4 Research Centre for Applied Science and Engineering, Universidad de Guadalajara, Guadalajara 44214, Mexico; mario.garcia@academicos.udg.mx; 5 Instituto de Ciencias Básicas e Ingeniería, Universidad Autonoma del Estado de Hidalgo, Pachuca 42111, Mexico; obed_perez@uaeh.edu.mx; 6 Faculty of Engeniering, Universidad Autonoma Nacional de Mexico, Ciudad de México 04510, Mexico; 7 Physics Department, Universidad del Atlantico, Barranquilla 081008, Colombia

**Keywords:** ZnO, nanostructures, thin films, pH control, solar cells, CBD

## Abstract

In this study, the impact of pH on the production of ZnO nanostructured thin films using chemical bath deposition was investigated for the purpose of enhancing the efficiency of solar cells. The ZnO films were directly deposited onto glass substrates at various pH levels during the synthesis process. The results indicate that the crystallinity and overall quality of the material were not affected by the pH solution, as observed through X-ray diffraction patterns. However, scanning electron microscopy revealed that surface morphology improved with increasing pH values, leading to changes in the size of the nanoflowers between pH 9 and 11 values. Furthermore, the ZnO nanostructured thin films synthesized at pH levels of 9, 10, and 11 were utilized in the fabrication of dye-sensitized solar cells. The ZnO films synthesized at pH 11 exhibited superior characteristics in short-circuit current density and open-circuit photo-voltage compared with those produced at lower pH values.

## 1. Introduction

The pH solution (ZnO) nanostructured thin films for solar cell applications are growing abruptly in the last two decades because of their wide direct band gap, principally. For example, high-efficiency solar cells require examination of how to manage the light effectively with new materials, geometries, structures, etc. ZnO nanostructured thin films are an interesting material because of their novel electronic structure and their mechanical, plas-monic, photonic, and electromechanical properties, among others. It is well known that the ZnO semiconductor band gap is between 3.2 to 3.4 eV; this property makes the ZnO semiconductor effective for many novel applications. In the last decade, more attention has been paid to building three-dimensional complex ZnO nanostructures, which not only inherit the excellent properties of the single building blocks but also provide potential applications in the bottom-up fabrication of functional devices. Furthermore, as the ZnO semi-conductor is not toxic, it was relatively environmentally friendly when we used it during the growth process. One significant phenomenon is the creation of ZnO nanostructures, which involves sequentially modifying primary structures. The pH values in the solution play a crucial role in this process. ZnO nanostructured thin films have been produced by several research groups worldwide, with a focus on their potential as efficient photocatalysts. The advantages of ZnO nanostructures, such as improved light harvesting, increased surface area, and porous structures, contribute to their appeal for such applications. For example, in ref. [1], the authors presented a review of the fabrication methods and growth mechanisms, and they paid particular attention to the photocatalytic applications of hierarchical ZnO nanostructures. Additionally, in ref. [2], an investigation on the formation of diverse ZnO nanostructures based on the solvent, temperature, and pH as adjustable parameters was presented. In ref. [3] the authors presented a study on the effect of different pH values, starting at pH 6, in an aqueous growth solution on the morphology, elemental chemical composition, crystal structure, and optical properties of ZnO nanostructures. This study was carried out using energy-dispersive analysis (EDX), UV-Visible Spectrometer, field emission scanning electron microscopy (FE-SEM), and X-ray diffraction (XRD). They observed that increasing pH values affected the influence intensity of the preferred orientation plane (002) and average transmittance spectrum. Additionally, they found that the crystal size fluctuated between 36.30 nm and 84.33 nm, with different pH values from 6.7 to 12. However, in ref. [4] it was indicated that the increase in the pH values of the solution leads significantly to a modification of the ZnO morphology from rod-like to prism-like and flower-like structures. In other words, refs. [3,4] showed that if we change the pH values, we can have some modifications in the ZnO nanostructures, including nanoflowers. However, which pH values are more important for fabricating ZnO nanostructured thin films, especially for increasing the efficiency of solar cells? In different works, the fabrication of diverse ZnO nanostructures with variations in the morphologies and dimensionalities has been achieved through different processing parameters, solvents, pH, and temperature [5,6,7,8]. In this work, we study the pH effect on the fabrication of ZnO nanostructured thin films by chemical bath deposition on increasing the efficiency of solar cells. We observed that the crystallinity and compositional analysis indicate that the pH solution does not affect the quality of the material. As pH values were changed with an increase from 9 to 11, the formation of excellent ZnO nanostructured thin films was unaffected. An increase in the band gap was found from 3.26 to 3.31 eV for pH 9 and pH 11, respectively.

## 2. ZnO Nanostructured Thin Films at Different pH Values

Zinc oxide (ZnO) is a semiconductor widely studied for its optical properties. Its gap is between 3.2 and 3.4 eV at room temperature, and the value reported here is between 3.2–3.31 eV. ZnO nanostructured thin films are used in solar cells, as a gas sensor, in a transducer, and as a transparent conductive electrode. In terms of obtaining it, it is very versatile since it can be prepared in bulk or in thin films. Different papers have presented that an important factor in fabricating high-quality devices based on ZnO is the effect of pH on the morphology and properties of ZnO. In these studies, the pH value in the solution appears to be a critical parameter for the particle size, the morphology of the structure, and phase formation during the solution method. For example, in ref. [5], the authors presented the preparation of ZnO nanostructures on SiO_2_-buffered Si substrates; here, the authors used various chemical precursors and different processing methods. In ref. [6], the authors presented a systematic study on varying the pH values of the precursor solution and analyzing the morphological variation of the ZnO nanostructure, where the zinc acetate dihydrate and sodium hydroxide were used as a precursor, which was refluxed at 90 °C for an hour. In ref. [7], the authors obtained the pompom-like ZnO nanostructures by using zinc nitrate hexahydrate and ammonia. In ref. [8], the authors presented the rose-like zinc oxide nanostructures by using ZnCl_2_ and ammonia (25%) synthesized through a hydrothermal decomposition method on a copper plate substrate. The effects of temperature on the optical and electrical properties of ZnO nanoparticles synthesized by the sol–gel method were presented in ref. [9]. The most recent results were presented in ref. [10], where the authors showed the effects of experimental configuration on the morphology of two-dimensional ZnO nanostructures synthesized by thermal chemical vapor deposition. Additionally, in ref. [11], the authors presented nano-/micro-structured ZnO rods synthesized by thermal chemical vapor deposition with a perpendicular configuration. In ref. [12], the authors presented the facet-selective morphology-controlled remote epitaxy of ZnO microcrystals via wet chemical synthesis. The applications found to be the most important regarding the effects of ZnO nanostructures on the performance of dye-sensitized solar cells were presented in refs. [13,14,15,16,17,18]. Furthermore, in ref. [14] the authors presented the zinc oxide nanostructures by chemical vapor deposition as anodes for Li-ion batteries.

For this work, we fabricated three different ZnO nanostructured thin films. These were obtained from a solution of ammonium hydroxide (NH_4_OH) and zinc sulfate (ZnSO_4_), controlling the pH value between 9 and 11 and the temperature between 85 and 100 °C. The precursor solution is zinc sulfate ZnSO_4_ 7H_2_O and Ammonia NH_3_ (25%) in a 1:10 molar ratio at room temperature, and the commercially available precursors of zinc sulfate (99.7% purity) and zinc nitrate (99.3% purity). The complexing agents used were ammonia (NH_3_) and ammonium hydroxide (NH_4_OH). All solutions were prepared with distilled water. Ammonium hydroxide was also used to adjust the pH. The substrates were glass sheets (slides) with dimensions 2 cm × 2 cm × 1 mm. These were washed with soap and water, then immersed in a mixture of sulfuric acid H_2_SO_4_ and distilled water at a ratio of 1:10 for 30 min, which was heated to around the boiling point. Subsequently, a rinse was carried out in ethanol and then acetone for 5 min. Finally, we obtained the formation of ZnO nanostructured thin films by chemical bath deposition with pH 9, pH 10, and pH 11 values. To assemble the dye-sensitized solar cells, we prepared thin film electrodes that were immersed in a dye solution at room temperature for one day, rinsed with anhydrous ethanol, and dried; this method has been reported in other literature [16,17,18,19].

## 3. Results

**Figure 1:**
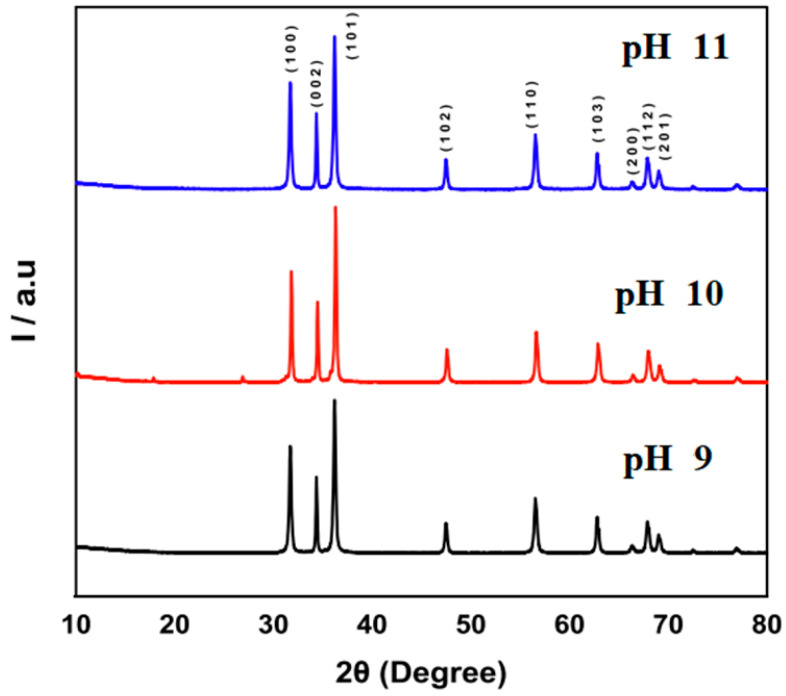
XRD patterns of ZnO with pH 9 (black), pH 10 (red) and pH 11 (blue).

In this section we present the results of the study of pH effect in the fabrication of ZnO nanostructured thin films by CBD. We used field-emission scanning electron microscopy (FE-SEM, JEOL, 15 kV) to analyze the morphology and chemical composition of the as-synthesized ZnO nanostructures. Additionally, Powder X-ray Diffraction (PXRD) patterns were recorded with a Rigaku XRD Ultima IV multipurpose diffractometer. Figure 1 shows the X-ray diffraction pattern; all detectable peaks can be indexed to the ZnO wurtzite structure. In the wurtzite lattice parameters, the values of *d* were calculated from Bragg’s equation [20], *nλ* = *2dsin θ*, where *n* is the order of diffraction (usually n = 1), λ is the X-ray wavelength, and *d* is the spacing between planes of given Miller indices *h*, *k* and *l.* These values are a function of increasing pH 9, pH 10, and pH 11 values. One can see that all the diffraction peaks in the pattern are well-matched in general with other studies already reported in the literature [8,9,10,11]. Additionally, the changes observed in the positions of (100), (002), and (101) reflections in X-ray diffraction (XRD) spectra between pH 11 and pH 9 can be attributed to a reduction in bond lengths [21]. This means we have excellent ZnO nanostructured thin films because the higher intensity and narrower spectral width of ZnO peaks in the spectrum indicate the good crystallinity of the product, and it can be used to fabricate high-quality semiconductor devices.

Zinc oxide nanostructures with a wurtzite structure are believed to form because of the anisotropic growth along the (001) plane, which experiences a faster growth rate under hydrothermal conditions. By controlling the pH values within the range of 9 to 11, it is possible to fabricate various types of nanostructures, such as nanorods, nanosheets, and nanoflowers. One can see a higher intensity of the (101) diffraction peak in all the patterns, further providing evidence of the preferential growth direction along the *c*-axis. The good crystallinity of the hierarchical ZnO nanostructures is revealed by strong and narrow peaks.

**Figure 2:**
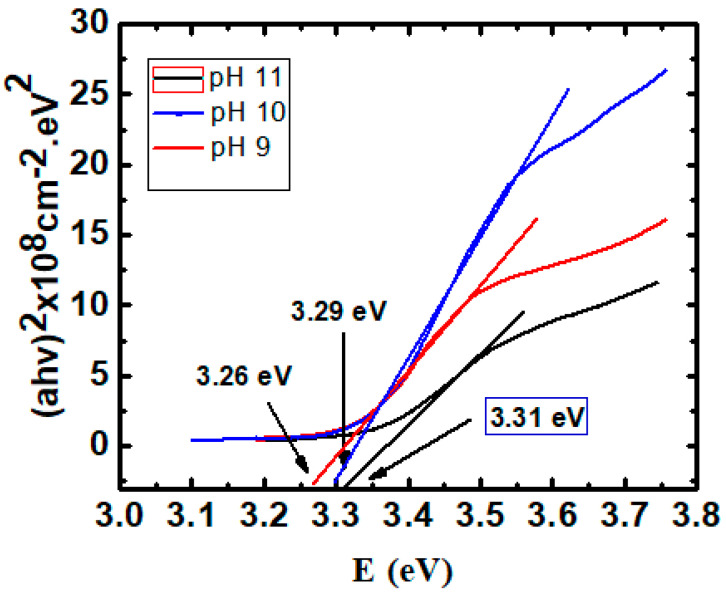
The plot of (*αhν*)^2^ versus (*hν*) of ZnO nanostructured thin films for pH 9, pH 10, and pH 11.

Figure 2 shows the plot of (*αhν*)^2^ versus (*hν*) for ZnO nanostructured thin films. One can see the band gap was found to be 3.26, 3.29, and 3.31 eV for pH 9, pH 10, and pH 11, respectively. Additionally, there is a higher band gap for pH 11, likely due to the presence of zinc hydroxide. However, the smaller band gap was found to be decreased to 3.26 eV for pH 9, which can be attributed to the lowest thickness and because of the removal of Zn (OH)_2_ particles from the surface of the film and/or the removal of defect levels. Nevertheless, in general, we have obtained the typical band gap values already reported in the literature [15].

**Figure 3:**
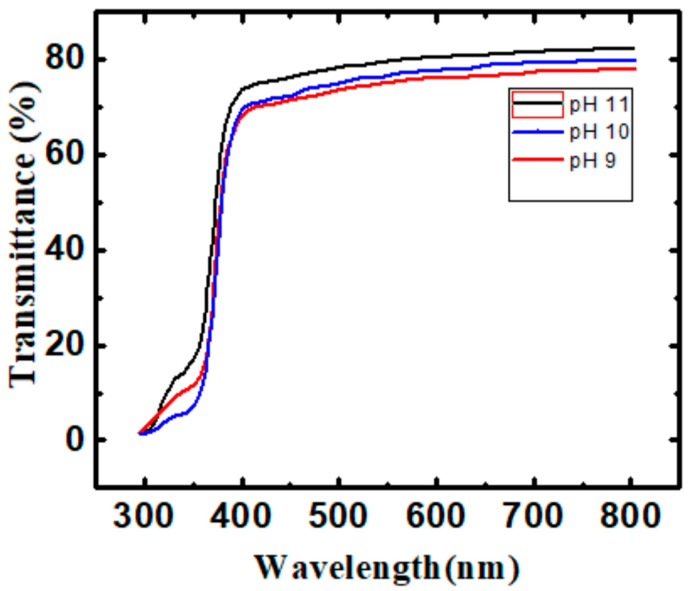
Transmittance of ZnO nanostructured thin films with wavelength (λ) for pH 9, pH 10, and pH 11.

The variation of the transmittance of the ZnO nanostructured thin films in three samples with a wavelength λ is shown in Figure 3. Here, one can see the variation of transmittance has increased, with an increase in the pH value, in this case, pH 11. This behavior can be attributed to the generation of extra energy levels after irradiation between the valance and conduction bands. These values are typical in this kind of research work using this method [12,13,14,15]. When the transmittance value of ZnO thin films is nearly up to 80%, that indicates the ZnO thin films have a good probability of transparent conducting oxide layer application, for example, in solar cell applications. Additionally, this tendency showed the improvement of the structure of the films caused by the pH 11 value.

**Figure 4:**
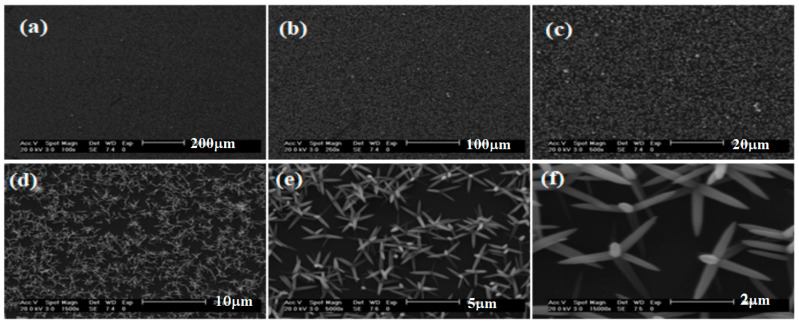
Typical FESEM images of the bunches (nanoflowers) of synthesized ZnO nanosheets at 85 °C for ZnO with pH 9 value at different scales: (**a**) 200 µm, (**b**) 100 µm, (**c**) 20 µm, (**d**) 10 µm, (**e**) 5 µm, (**f**) 2 µm.

Figure 4 shows the typical FESEM images of nanoflowers of synthesized ZnO nanostructured thin films at 85 °C and a pH 9 value. Here, one can see the ZnO nanostructures composed of nanosheets. From the image at higher magnification (Figure 4e), it is observed that each ball has a flower-like nanostructure with an average diameter of 140 nm. From the enlarged image (Figure 4f), one can see that each nanoflower is composed of nanosheets, spreading in all possible directions. The size of the nanosheets is around 140 nm, approximately. With these sizes, the ZnO nanoflower can be found in solar cell applications because of their stability and as an antimicrobial agent for water remediation [15]. Additionally, the ZnO nanoflower has been used as a therapeutic agent for many diseases and in the designing of biosensors [15].

**Figure 5:**
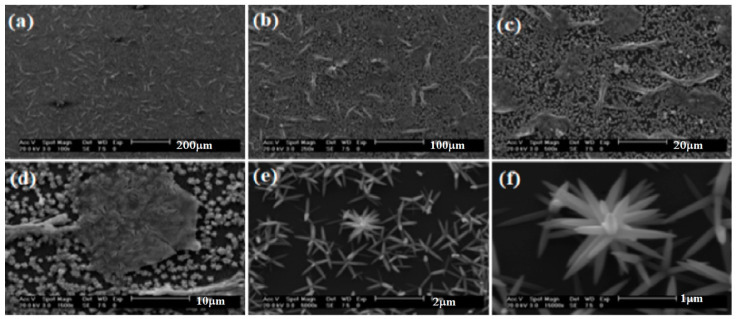
Typical FESEM images of the bunches (nanoflowers) of synthesized ZnO nanosheets at 85 °C for ZnO with pH 10 at different scales: (**a**) 200 µm, (**b**) 100 µm, (**c**) 20 µm, (**d**) 10 µm, (**e**) 2 µm, (**f**) 1 µm.

Figure 5 shows the typical FESEM images of the nanoflowers of synthesized ZnO nanosheets at 85 °C and pH 10. In this case, the structures composed of nanosheets were observed at all of the temperatures too. The nanoflowers can be seen with the same dimensions as with pH 9. However, here we can observe different nanoflower forms; see Figure 5e. For example, in Figure 5f, we can see the nanoflower with more nanosheets.

**Figure 6:**
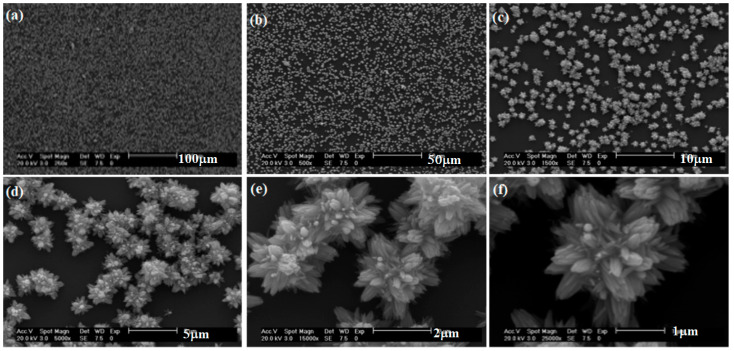
Typical FESEM images of the bunches (nanoflowers) of synthesized ZnO nanosheets at 85 °C for ZnO with pH 11 at different scales: (**a**) 100 µm, (**b**) 50 µm, (**c**) 10 µm, (**d**) 5 µm, (**e**) 2 µm, (**f**) 1 µm.

Figure 6 shows the typical FESEM images of the nanoflowers of synthesized ZnO nanosheets at 85 °C and pH 11. Here, we can see nanoflowers of various sizes. The nanoflowers can be observed with the same dimensions as with pH 10. However, here we can also see different nanoflower forms; see Figure 6d–f. For example, in Figure 6f, we can see the nanoflower with more nanosheets.

## 4. Discussion

In different studies, ZnO nanostructured thin films have several morphologies, but in this study, we focus on nanosheets to form nanoflowers. The development of nanoflowers can be broken down into two stages: firstly, the creation of spherical molecules through nucleation, followed by their growth into flower-shaped structures. In the case of an aqueous solution of zinc salts, compounds with low solubility are produced when the solution reacts. The morphology of the resulting zinc oxide nanostructures can be manipulated by utilizing different salts and adjusting the processing variables, including the temperature, quantity of the salt used, and the pH level of the solution. This is a well-established technique for controlling the structural characteristics of zinc oxide nanostructures [6]. These ZnO nanostructured thin films can be used to concentrate light, and they can increase the efficiency of solar cells and other applications. In this case, the effect of the pH level on ZnO nanostructured thin films was analyzed to increase the efficiency of solar cells. ZnO nanostructured thin films with pH levels 10 and 11 have the best ZnO nanostructures. The development of novel ZnO nanostructures by CBD can lead to excellent nanoflowers or nanosheets with multiple potential applications in different areas, such as in the fields of sensors, photoelectrochemical devices, high-efficiency solar cells, and devices. Additionally, we found that the pH does not greatly affect the crystallinity and compositional analysis; this can be observed in the analysis from the X-ray diffraction pattern.

**Figure 7:**
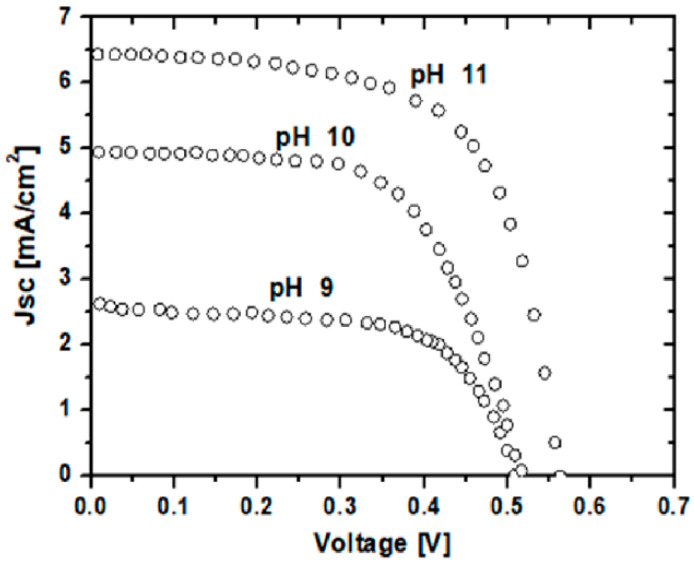
J-V curves of nanostructure-based dye-sensitized solar cells.

Figure 7 shows the photocurrent–voltage curves of the dye-sensitized solar cell assembled using ZnO nanostructured thin films. A dye-sensitized solar cell assembled with ZnO nanostructured thin films prepared at pH 9 had a *V*oc of 0.52 V and a *J*sc of 2.6 mA/cm^−2^. For pH 10, it had a *V*oc of 0.53 V and a *J*sc of 4.8 mA/cm^−2^. For pH 11, it had a *V*oc of 0.56 V and a *J*sc of 6.4 mA/cm^−2^. It is observed that dye-sensitized solar cells using ZnO nanostructured thin films prepared at pH 11 showed higher short-circuit current density (*J*sc) and conversion efficiency compared with the solar cells prepared using ZnO nanostructured thin films prepared at pH 9. The reason for the higher *J*sc is the formation of small nanoflower-like structures on the surface; it may increase the dye absorption area. The higher current density and overall conversion efficiency originated from the improvement of the dye-absorption area and the light harvesting [19].

## 5. Conclusions

The present investigation has been carried out to optimize the pH level to produce excellent ZnO nanostructured thin films by the CVD method. Additionally, this work shows the advances in the solution phase synthesis to fabricated nanoflowers assembled by nanosheets. The results are very important because these ZnO nanostructured thin films can be found in applications in environmental and biomedical fields, such as lithium-ion batteries, photocatalysis, electrochemical sensors, and biomedical sensors, although in this work, they increased the efficiency of solar cells. We found that the pH solution does not affect the crystallinity of hierarchical ZnO nanostructures, as observed from the X-ray diffraction pattern. However, the small spherical sheets of micro-flower composed of the sheet structure were changed with increased pH values from 9 to 11. We can conclude that pH 11 is ideal for creating nanoflowers; however, these nanoflowers can also be found at the pH 9 value.

## Data Availability

Not applicable.
